# Integrative Transcriptomic and Machine-Learning Analysis Reveals Immune-Inflammatory and Stress-Response Alterations in MRONJ

**DOI:** 10.3390/ijms262411788

**Published:** 2025-12-05

**Authors:** Galina Laputková, Ivan Talian, Ján Sabo

**Affiliations:** Department of Medical and Clinical Biophysics, Faculty of Medicine, Pavol Jozef Šafárik University, 040 11 Košice, Slovakia; galina.laputkova@upjs.sk (G.L.); jan.sabo@upjs.sk (J.S.)

**Keywords:** MRONJ, transcriptomics, immune dysregulation, stress response, WGCNA, biomarkers

## Abstract

Medication-related osteonecrosis of the jaw (MRONJ) is a serious adverse effect of antiresorptive and antiangiogenic therapies, yet its molecular mechanisms remain poorly defined. The present study employed an analysis of microarray data (GSE7116) from peripheral blood mononuclear cells of patients with multiple myeloma, myeloma patients with MRONJ, and healthy controls. Differentially expressed genes were identified using the limma package, followed by functional enrichment analysis, weighted gene co-expression network analysis, and LASSO regression and CytoHubba network ranking. The predictive performance was validated by means of nested cross-validation, Firth logistic regression, and safe stratified 0.632+ bootstrap ridge regression. The profiling revealed distinct gene expression patterns between the groups: the upregulation of ribosomal and translational pathways, as well as the suppression of neutrophil degranulation and antimicrobial defense mechanisms, and identified key candidate genes, including *PDE4B*, *JAK1*, *ETS1*, *EIF4A2*, *FCMR*, *IGKV4-1*, and *XPO7*. These genes demonstrated substantial discriminatory capability, with an area under the curve ranging from 0.95 to 0.99, and were found to be functionally linked to immune system dysfunction, cytokine signaling, NF-κB activation, and a maladaptive stress response. These findings link MRONJ to systemic immune-inflammatory imbalance and translational stress disruption, offering novel insights and potential biomarkers for diagnosis and risk evaluation.

## 1. Introduction

The 2022 American Association of Oral and Maxillofacial Surgeons (AAOMS) policy paper defines medication-related osteonecrosis of the jaw (MRONJ) based on the following criteria: no history of radiation therapy to the jaws or evident metastatic disease to the jaws, exposed bone or bone that can be probed through an intraoral or extraoral fistula in the maxillofacial region that has persisted for more than eight weeks, and current or past treatment with antiresorptive and/or antiangiogenic agents [1]. Antiresorptive agents (e.g., bisphosphonates and denosumab) are widely used to treat osteoporosis, to control hypercalcemia and skeletal pain from bone metastases, and as part of therapy for multiple myeloma, either as a standalone treatment or in combination with antiangiogenic or immunomodulatory agents [1]. At present, due to the influence of pharmaceutical agents targeting the osteoclastogenesis process, patients suffering from a variety of pathologies—including, but not limited to, giant cell tumors, rheumatoid arthritis, Paget’s disease of bone, fibrous dysplasia, and osteogenesis imperfecta—require these pharmaceutical agents to manage their respective conditions [2]. As a result, the number of individuals susceptible to the development of MRONJ has increased significantly. The impact of MRONJ on quality of life is substantial [3], particularly in advanced cases characterized by severe pain and impaired oral intake. In patients with oncologic diseases, the condition has the potential to be life-threatening. Consequently, elucidating the pathophysiology of MRONJ is imperative to enhance prevention and treatment strategies. Even though research on MRONJ has been ongoing since its initial report in 2003 [4], the precise etiology of the condition remains uncertain, and a universally accepted treatment plan is yet to be established. The pathogenesis of MRONJ is complex, involving an interacting array of genetic, metabolic, and immunological factors [5]. A multitude of hypotheses have been proposed to explain the development of MRONJ. However, the predominant hypothesis suggests that it is caused by excessive suppression of bone metabolism and remodeling triggered by anti-resorptive medications [6]. The anti-resorptive agent in question exerts its effect by inhibiting both the process of osteoclastic bone resorption and that of osteoblastic bone formation. This effect arises from an interruption in the coupling of osteoblasts and osteoclasts. As posited in the research by Kim, J.-M. et al. [7], anti-resorptive agents have been shown to reduce the release of osteogenic clastic factors by inhibiting osteoclast differentiation. In addition to this, the release of osteogenic growth factors embedded within the bone matrix is also reduced by anti-resorptive agents as a result of decreased bone resorption. Researchers also deliberate on hypotheses that encompass oral infection [8] and inflammation [9]. The antiangiogenic impact and soft tissue toxicity of bisphosphonates are also assessed [10]. The discussion concludes with a focus on the inhibition of lymphangiogenesis [11]. Osteonecrosis is a condition characterized by the death of local bone and marrow cells due to continuous stimulation by detrimental factors, resulting in aberrant osteoimmune function [12]. This phenomenon leads to the occurrence of a chronic inflammatory microenvironment that inhibits bone regeneration and repair. In a plethora of bone tissue diseases, innate immune cells and adaptive immune cells interact with bone cells. The effects of this interaction on bone metabolic homeostasis have attracted increasing attention, resulting in the establishment of a new field: osteoimmunology. A thorough examination has been carried out to identify the regulatory mechanisms that control the inflammatory response, revealing that immune cells are the principal components involved. As a result, osteoimmune dysfunction has attracted considerable interest from the scientific community as a potential causative factor of osteonecrosis [13]. The onset of MRONJ and the activity of particular genes may be related, according to earlier research. Scientists analyzed gene expression data from individuals with multiple myeloma derived from microarray experiments and identified seven hub genes (*ACTB*, *FN1*, *GAPDH*, *JUN*, *PTPRC*, *STAT3*, and *TNF*) linked to bisphosphonate-induced osteonecrosis of the jaw [14]. However, dysregulation of *GAPDH* expression in MRONJ in patients with multiple myeloma should be approached with caution, as *GAPDH* is implicated in multiple cancer-related biological processes, having recently emerged as a primary focus in cancer research [15,16]. The preceding analyses resulted in the identification of three salivary proteins (*AACT*, *HBD*, and *MMP9*) [17]; 17 key genes; and hsa-mir-16-1, hsa-mir-21, hsa-mir-23a, hsa-mir-145, hsa-mir-186, hsa-mir-221, and hsa-mir-424 [18] associated with MRONJ.

Despite the noteworthy advancements in elucidating the mechanisms underlying MRONJ, challenges persist with regard to the early diagnosis and treatment of this condition due to its heterogeneous radiological manifestations and unpredictable course [19].

Traditional diagnostic methods often prove ineffective, limiting their ability to provide timely diagnosis, treatment, and prognosis for patients. Genetic testing can serve as an important tool in the diagnosis and treatment of MRONJ, increasing the accuracy and effectiveness of both diagnostic and therapeutic approaches [20]. The identification of genetic markers associated with MRONJ will enable earlier and more accurate diagnosis, facilitating timely treatment and improving patient care. Genetic screening tests have therefore become a key element of therapeutic practice, increasing the accuracy of diagnosis and facilitating personalized treatment strategies. Identifying disease-associated genes via genetic testing, coupled with the verification of relevant biomarkers, would enhance the speed and efficiency of MRONJ diagnosis and treatment, thereby improving both short-term clinical outcomes and long-term disease management.

This study will investigate genes, biomarkers, and molecular processes related to MRONJ through comprehensive bioinformatics analysis to improve MRONJ diagnosis.

## 2. Results

### 2.1. Screening Analysis of the DEGs

To investigate the genes associated with the onset of MRONJ, a retrospective analysis was conducted using PBMC expression profiles from the GSE7116 dataset. This dataset included samples from 11 MM_ONJ patients, 10 age-matched MM controls, and 5 Healthy controls.

**Figure 1:**
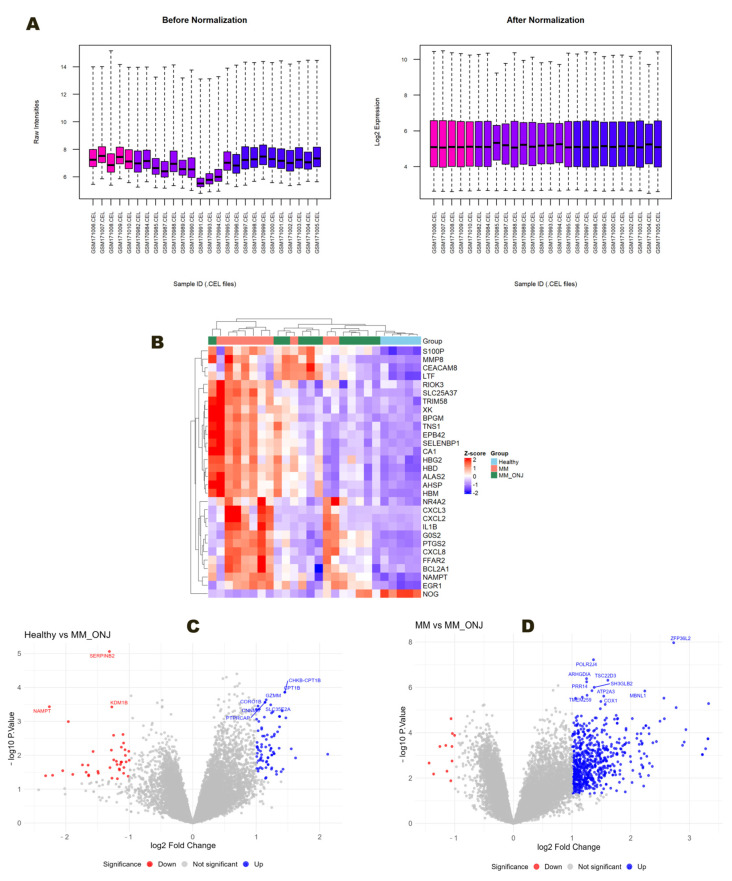
Screening analysis of DEGs. (**A**) Box plots of the distribution and normalization of raw expression data across samples. Pink—Healthy, Violet—MM_ONJ, Blue—MM. (**B**) Heatmap of the expression patterns of the top 30 DEGs. (**C**) Volcano plot of significantly upregulated and downregulated genes, Healthy vs. MM_ONJ. (**D**) Volcano plot of significantly upregulated and downregulated genes MM vs. MM_ONJ. The *X*-axis represents log_2_ fold change, and the *Y*-axis shows −log_10_(*p*-value). Groups: Healthy—healthy volunteers; MM—multiple myeloma patients without MRONJ; MM_ONJ—multiple myeloma patients with MRONJ. A positive log_2_FC indicates genes upregulated in Healthy relative to MM_ONJ (MM relative to MM_ONJ). A negative log_2_FC indicates genes downregulated in Healthy relative to MM_ONJ (MM relative to MM_ONJ).

Raw microarray data were normalized and preprocessed for downstream analysis (Figure 1A). Differential gene expression analysis identified 22,173 transcripts per comparison. Using a significance threshold of *p* < 0.05 and |log_2_ fold change| > 1, a total of 124 DEGs were identified in the Healthy vs. MM_ONJ comparison, and 426 DEGs in the MM vs. MM_ONJ comparison (Supplementary Materials: S1A, S1B). A visual representation of these findings was achieved using a heat map, which highlights the top DEGs (Figure 1B). Furthermore, the implementation of volcano plots (Figure 1C,D) elucidated genes that exhibited significant upregulation, in contrast to those that demonstrated significant downregulation.

### 2.2. Gene Set Enrichment Analysis

**Figure 2:**
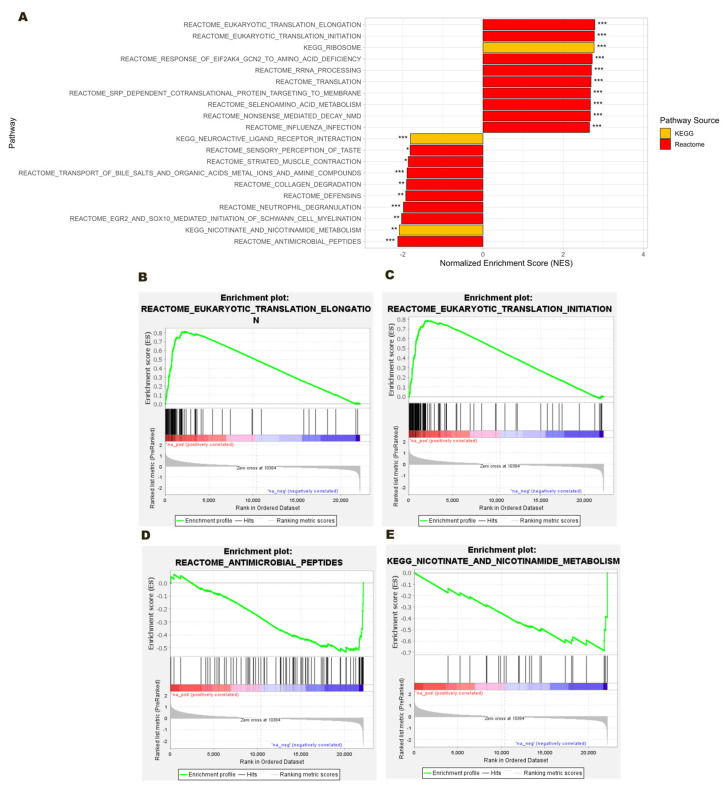
GSEA of Reactome and KEGG pathway enrichment in the Healthy vs. MM_ONJ group. (**A**) Bar plot displaying the top ten upregulated and downregulated pathways ranked by NES. (**B**) Enrichment plot for significantly upregulated pathway: Eukaryotic Translation Elongation. (**C**) Enrichment plot for significantly upregulated pathway: Eukaryotic Translation Initiation. (**D**) Enrichment plot for significantly downregulated pathway: Antimicrobial Peptides. (**E**) Enrichment plot for significantly downregulated pathway Nicotinate and Nicotinamide Metabolism. The running enrichment score for the gene set (green curve), with the ranked gene list indicated below. Genes are ordered from most upregulated (left, red) to most downregulated (right, blue), and vertical black lines represent hits for genes within the pathway. Significance thresholds: *** *adj. p* < 0.001, ** *adj. p* < 0.01, * *adj. p* < 0.05.

GSEA was performed for both distinct comparisons, Healthy vs. MM_ONJ and MM vs. MM_ONJ, to gain more profound insight into the biological pathways associated with the expression of genes related to MRONJ. In the Healthy vs. MM_ONJ comparison, the DEGs were significantly enriched in those pathways that were related to protein synthesis and ribosomal function (Figure 2). The top five upregulated pathways (based on positive Normalized Enrichment Score, NES, and *p* < 0.001) included Eukaryotic Translation Elongation, Eukaryotic Translation Initiation, Ribosome, Response of *EIF2AK4* (*GCN2*) to Amino Acid Deficiency, and rRNA Processing.

On the other hand, the top five downregulated pathways in the Healthy vs. MM_ONJ group (Figure 2) were primarily associated with innate immune defense, in particular neutrophil and antimicrobial activities. These included Antimicrobial Peptides, Nicotinate and Nicotinamide Metabolism, *Egr2* and *SOX10*-Mediated Initiation of Schwann Cell Myelination, Neutrophil Degranulation, and Defensins. Our findings indicate that for multiple myeloma patients with MRONJ disease, a significant suppression of neutrophil activity and host defense mechanisms can be observed.

**Figure 3:**
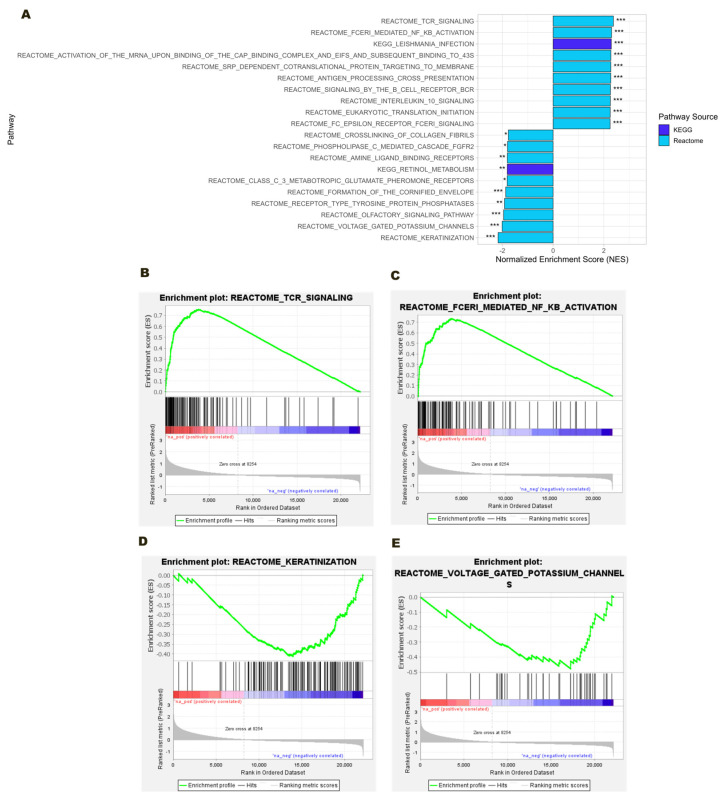
GSEA of Reactome and KEGG pathway enrichment in the MM vs. MM_ONJ group. (**A**) Bar plot displaying the top ten upregulated and downregulated pathways based on NES. (**B**) Enrichment plot for significantly upregulated pathway: TCR Signaling. (**C**) Enrichment plot for significantly upregulated pathway: FcεRI-Mediated NF-κB Activation. (**D**) Enrichment plots for significantly downregulated pathways: Keratinization. (**E**) Enrichment plot for significantly downregulated pathway: Voltage-Gated Potassium Channels. The running enrichment score for the gene set (green curve), with the ranked gene list displayed below. Genes are ordered from most upregulated (left, red) to most downregulated (right, blue). Vertical black lines indicate positions of genes in the tested gene set. Significance thresholds: *** *adj. p* < 0.001, ** *adj. p* < 0.01, * *adj. p* < 0.05.

For the MM vs. MM_ONJ comparison (Figure 3), both up- and downregulated pathway signatures were observed. The top five significantly upregulated pathways included T Cell Receptor (TCR) Signaling, FcεRI-Mediated NF-κB Activation, Leishmania Infection, Activation of mRNA upon Binding of the Cap-Binding Complex and eIFs, and Subsequent Binding to 43S, SRP-Dependent Cotranslational Protein Targeting to Membrane. At the same time, the five pathways with the most significant downregulation were: Keratinization, Voltage-Gated Potassium Channels, Olfactory Signaling Pathway, Receptor-Type Tyrosine–Protein Phosphatases, and Formation of the Cornified Envelope. All pathways mentioned above exhibited statistically significant negative NES and *adj. p* < 0.05, with many reaching thresholds of *adj. p* < 0.001, thereby reinforcing their potential biological relevance to MRONJ pathogenesis.

### 2.3. Weighted Gene Co-Expression Network Analysis

**Figure 4:**
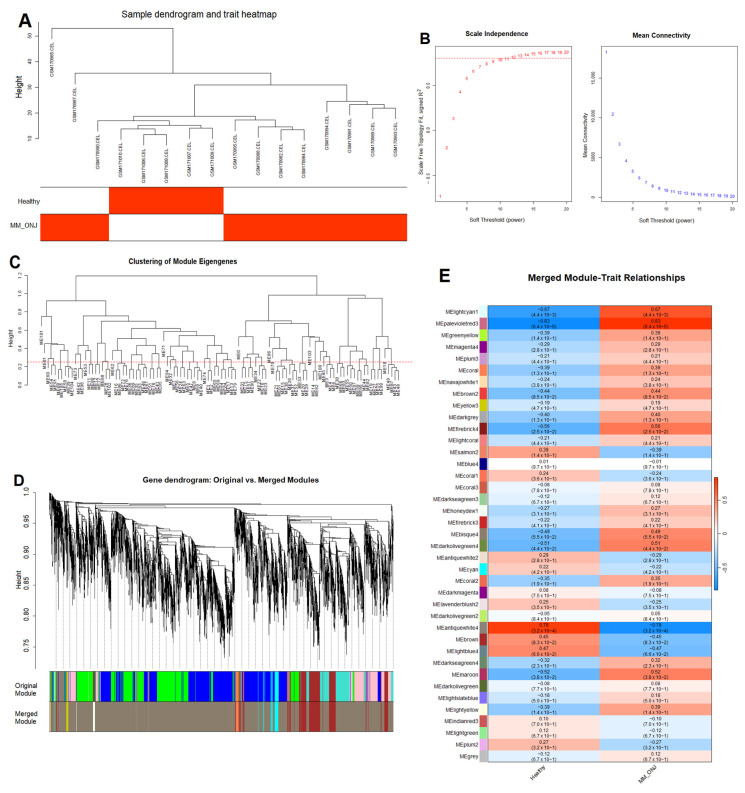
Co-expression network analysis of DEGs in the Healthy vs. MM_ONJ group using WGCNA. (**A**) Sample clustering dendrogram identifying potential outliers. (**B**) Selection of the soft-thresholding power based on scale-free topology fit (R^2^) and mean connectivity. The red dotted line represents the target scale-free topology fit threshold, R^2^ = 0.85. (**C**) Gene dendrogram with modules identified by dynamic tree cutting and merged at a cut height of 0.25 (the red line). (**D**) Cluster dendrogram displaying initial and merged modules, with distinct colors representing different co-expression modules. (**E**) Module-trait relationship heatmap: each row represents an ME, and each column represents a clinical trait. Each cell shows the Pearson correlation coefficient and the corresponding *p*-value. DEGs—differentially expressed genes; ME—module eigengene; Healthy—healthy volunteers; MM_ONJ—MM patients with MRONJ.

**Figure 5:**
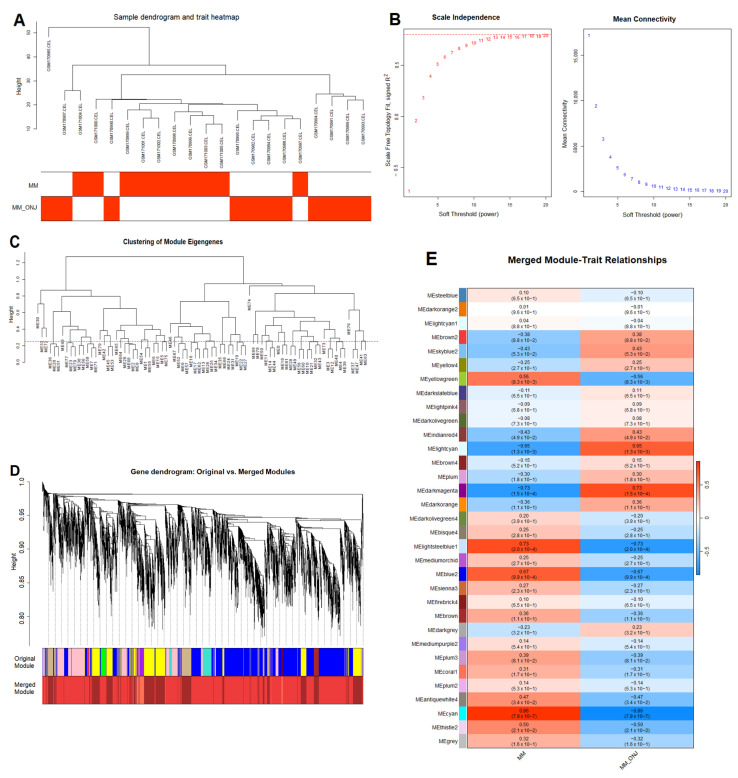
Co-expression network analysis of DEGs in the MM vs. MM_ONJ group using WGCNA. (**A**) Sample clustering dendrogram identifying potential outliers. (B) Selection of the soft-thresholding power based on scale-free topology fit (R^2^) and mean con-nectivity. The red dotted line represents the target scale-free topology fit threshold, R^2^ = 0.85. (**C**) Gene dendrogram with modules identified by dynamic tree cutting and merged at a cut height of 0.25 (the red line). (**D**) Cluster dendrogram displaying initial and merged modules, with distinct colors representing different co-expression modules. (**E**) Module-trait relationship heatmap: each row represents an ME, and each column represents a clinical trait. Each cell shows the Pearson correlation coefficient and corresponding *p*-value. DEGs—differentially expressed genes; ME—module eigengene; MM—multiple myeloma patients; MM_ONJ—MM patients with MRONJ.

WGCNA is a systems biology method that operates under the assumption that genes exhibiting similar expression profiles may often be functionally linked, co-regulated, or involved in the same biological pathways. This approach facilitates the establishment of a gene similarity network, which can subsequently be utilized to identify clusters comprising genes that exhibit a high degree of correlation. Subsequently, these genes are organized into modules through a hierarchical clustering process. Module eigengenes (MEs), defined as the first principal component of each module, were computed and correlated with binary clinical traits (Healthy vs. MM_ONJ; MM vs. MM_ONJ). A sample dendrogram was generated for each comparison to visualize relationships among samples (Figure 4A and Figure 5A). To determine the appropriate soft-thresholding power (β) for scale-free network topology, values of β = 15 (for Healthy vs. MM_ONJ) and β = 13 (for MM vs. MM_ONJ) were selected based on scale-free topology fit indices (Figure 4B and Figure 5B). For the Healthy vs. MM_ONJ comparison, the soft-thresholding power β = 15 was selected because it provided a stable, high scale-free topology fit (R^2^ plateau), reduced sensitivity to noise, and more biologically interpretable co-expression modules while simultaneously maintaining an acceptable mean connectivity. For the MM vs. MM_ONJ dataset, no soft-thresholding power reached the conventional R^2^ ≈ 0.85, likely due to limited sample size and weaker global co-expression structure. In such situations, the power that best approaches scale-free topology while maintaining stable connectivity was chosen. Therefore, β = 13 was selected, which showed the highest attainable R^2^, the most stable connectivity pattern, and minimal noise—representing the optimal practical compromise when an ideal scale-free fit cannot be achieved. Network construction and module detection were carried out using the following parameters: minModuleSize = 20 and mergeCutHeight = 0.15. Modules with eigengene correlations greater than 0.75 were merged, resulting in 39 and 31 merged modules for the two comparisons, respectively (Figure 4C and Figure 5C). Both raw and merged module dendrograms were visualized (Figure 4D and Figure 5D), with modules color-coded for ease of distinction. According to ME-trait heatmap analysis, four modules showed statistically significant associations with the onset of MRONJ in the Healthy vs. MM_ONJ comparison: MEantiquewhite4, MEpalevioletred3, MEfirebrick4, and MElightcyan1 (Figure 4E), comprising a total of 1261 genes. In the MM vs. MM_ONJ comparison, five modules were significantly associated with MRONJ: MElightcyan, MEcyan, MEdarkmagenta, MElightsteelblue1, and MEskyblue2 (Figure 5E), comprising 2512 genes in total. Modules exhibiting the strongest correlations with disease status were selected for downstream analysis.

### 2.4. Integrated CytoHubba-Identified PPI Hubs and LASSO-Selected Predictive Genes

**Figure 6:**
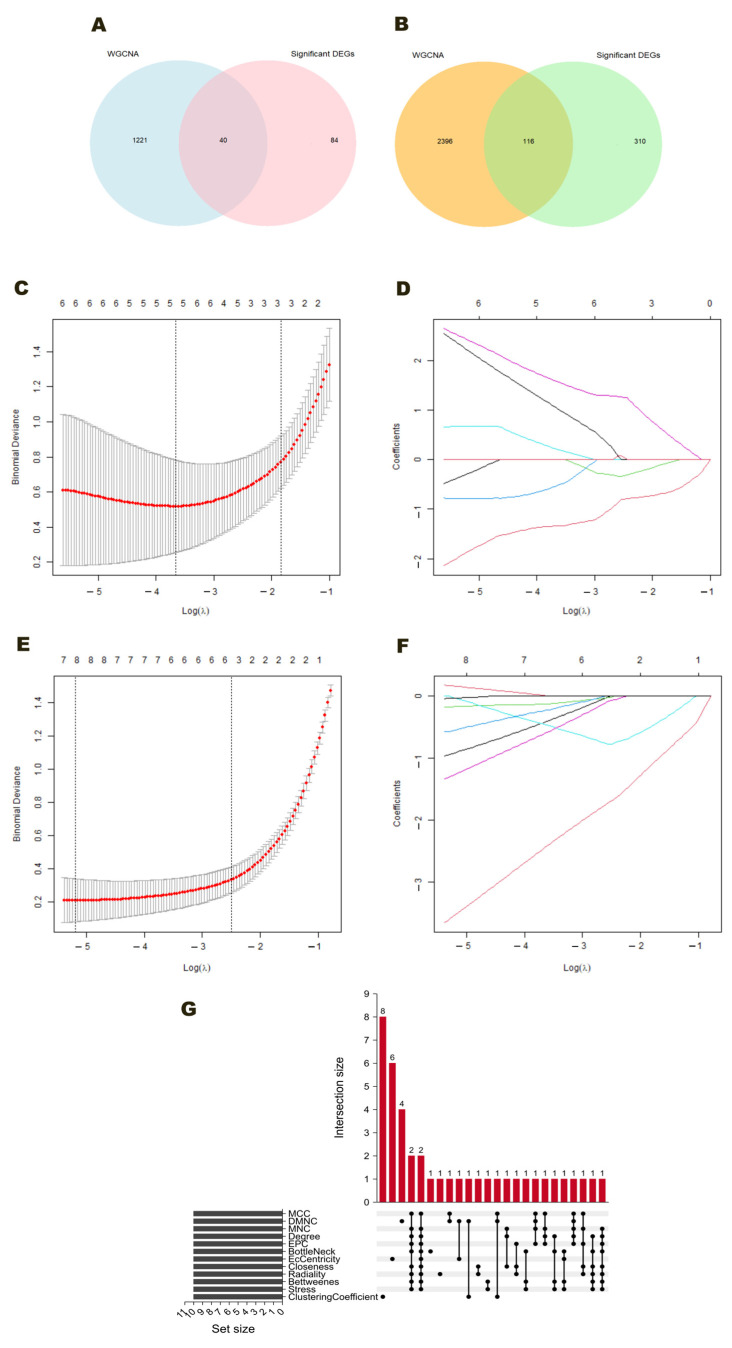
Screening of MRONJ-related DEGs based on LASSO regression and CytoHubba-identified PPI hubs. (**A**) Venn diagram showing the 40 disease-related hub DEGs obtained from the intersection between WGCNA-derived hub genes and significant DEGs for Healthy vs. MM_ONJ. (**B**) Venn diagram showing the 116 disease-related hub DEGs obtained from the intersection between WGCNA-derived hub genes and significant DEGs for MM vs. MM_ONJ. (**C**,**E**) Coefficient variation plots for variables in the LASSO regression models for Healthy vs. MM_ONJ and MM vs. MM_ONJ, respectively. (**D**,**F**) Ten-fold CV curves used to determine the optimal λ value in the LASSO regression models for Healthy vs. MM_ONJ and MM vs. MM_ONJ, respectively. Each colored line represents the coefficient trajectory of a single gene (or predictor) as the regularization parameter λ changes. (**G**) UpSet plot visualizing the results of 12 CytoHubba algorithms in Cytoscape (v 3.10.4) and the hub genes identified in the PPI network. The *x*-axis represents the overlap patterns among different algorithms, and the *y*-axis indicates the number of genes shared by each overlap combination.

In the Healthy vs. MM_ONJ comparison, a total of 40 hub module DEGs were identified by intersecting the 1261 hub module genes from four WGCNA-derived modules with the 124 DEGs from the previous analysis (significance threshold: *p* < 0.05 and |log_2_ fold change| > 1) (Figure 6A). In the MM vs. MM_ONJ comparison, 116 DEGs were identified by intersecting the 2512 hub genes from five WGCNA-derived modules with the 426 DEGs from the earlier screening (Figure 6B). The gene selection process was performed independently using two complementary approaches: LASSO regression and CytoHubba-based PPI network analysis, applied to both sets of hub genes under investigation.

In LASSO regression, λ_min_ corresponds to the regularization parameter that minimizes CV error, while λ_1Se_ is the largest value within one standard error of this minimum, favoring simpler models with fewer predictors. For the Healthy vs. MM_ONJ comparison, LASSO regression with 10-fold CV was performed (Figure 6C,D). The optimal λ values were λ_min_ ≈ e^−4.1^ and λ_1Se_ ≈ e^−3.1^. At λ_min_, six non-zero coefficients were retained. When using λ_1Se,_ a more parsimonious model with five non-zero coefficients (*FCMR*, *ZAP70*, *HBG2*, *ADM*, *NAMPT*) was obtained. Subsequently, the parameter λ_1Se_ was selected for further analyses to optimize the balance between model simplicity and its predictive accuracy. For the MM vs. MM_ONJ comparison, LASSO regression with 10-fold CV was also performed (Figure 6E,F). The optimal λ values were λ_min_ ≈ e^−4.8^ and λ_1Se_ ≈ e^−3.1^. At the minimum value of the parameter λ, eight non-zero coefficients were retained. The parameter λ_1Se_ yielded a more parsimonious model with five non-zero coefficients (*IGKV4-1*, *PPP6R2*, *XPO7*, *BTG2*, *PDE4B*). As was the case in the preceding comparison, the value of the parameter λ_1Se_ was selected in order to prioritize the simplicity of the model while ensuring the maintenance of a high degree of predictive performance. The network topology of the 40 and 116 DEGs identified in both comparisons was analyzed using the CytoHubba plugin in Cytoscape. Highly connected hub genes were extracted from the main complex DEG network using CytoHubba local-based methods—Maximum Neighborhood Component (MNC), Maximal Clique Centrality (MCC), Density of Maximum Neighborhood Component (DMNC), and Degree (Deg)—as well as global-based methods, including Betweenness Centrality (BC), BottleNeck (BN), Closeness Centrality (Clo), EcCentricity (EC), Edge Percolated Component (EPC), Stress (Str), and Radiality (Rad). From this analysis, 16 genes were identified that appeared in the intersection of at least three ranking methods: *BCL2L1*, *BCL10*, *CCL3*, *CD44*, *CREB1*, *CXCL1*, *CXCL3*, *EIF4A2*, *ETS1*, *EZR*, *IFIH1*, *IL1B*, *JAK1*, *PPP1R15A*, *TNFAIP3*, and *ZAP70* (Figure 6G).

### 2.5. Gene Ontology and Pathway Enrichment

**Figure 7:**
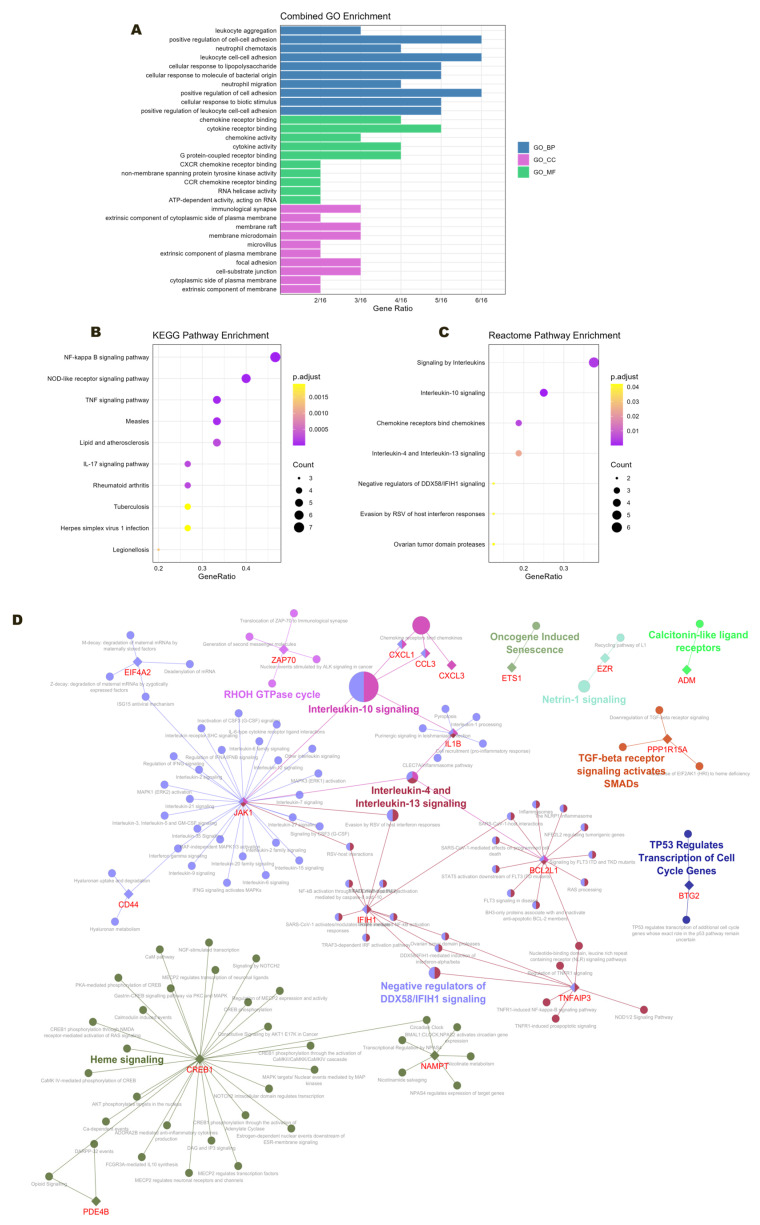
Gene ontology and pathway enrichment of 25 hub genes. (**A**) Bar plot of the combined GO enrichment analysis. (**B**) KEGG pathway enrichment. (**C**) Reactome pathway enrichment. (**D**) ClueGO/CluePedia network representation integrating KEGG and Reactome pathway enrichment, highlighting specific gene interactions. Network connectivity is represented by functional nodes and edges, which are shared between DEGs with a kappa score of 0.4. Only significant GO terms (*p* < 0.05) are displayed. Node size reflects *p*-value significance, node color indicates the specific pathways involved, and bold font denotes the most important functional GO terms defining each group. The names of DEGs involved in each group are shown in red font.

GO enrichment analysis was performed on the integrated set of 25 DEGs obtained from the intersection of CytoHubba and LASSO results (*BCL2L1*, *ADM*, *BCL10*, *BTG2*, *CCL3*, *CD44*, *CREB1*, *CXCL1*, *CXCL3*, *EIF4A2*, *ETS1*, *EZR*, *FCMR*, *HBG2*, *IFIH1*, *IGKV4-1*, *IL1B*, *JAK1*, *NAMPT*, *PDE4B*, *PPP1R15A*, *PPP6R2*, *TNFAIP3*, *XPO7*, *ZAP70*) using the BiNGO plugin in Cytoscape. The Benjamini–Hochberg false discovery rate method, with a significance level of *p* < 0.05, was employed to correct for the multiple testing. The three distinct domains: biological process (GO_BP), molecular function (GO_MF), and cellular component (GO_CC) were implemented. The selection of these domains was made to provide illustrative examples of the functional roles and regulatory context of the identified gene sets (Figure 7A). The top three enriched GO_BP terms, ranked by adjusted *p*-value, were as follows: GO:0070486, leukocyte aggregation; GO:0022409, positive regulation of cell–cell adhesion; and GO:0030593, neutrophil chemotaxis. The following key terms were identified for GO_MF: GO:004237, chemokine receptor binding; GO:0005126, cytokine receptor binding; and GO:0008009, chemokine activity. In GO_CC, the top three terms were classified as such: GO:0001772, immunological synapse; GO:0031234, extrinsic component of cytoplasmic side of plasma membrane; and GO:0045121, membrane raft. Pathway enrichment analysis of the integrated set of DEGs, performed using both KEGG and Reactome databases, revealed the biological relevance of the hub genes within the following signaling and metabolic pathways: KEGG: hsa04064 NF-kappa B signaling pathway, hsa04621 NOD-like receptor signaling pathway, hsa04668 TNF signaling pathway; Reactome: R-HSA-6783783 Interleukin-10 signaling, R-HSA-449147 Signaling by interleukins, R-HSA-380108 Chemokine receptors bind chemokines. All pathways met the significance threshold of *p* < 0.05 (Figure 7B,C). In parallel, a combined KEGG and Reactome pathway enrichment analysis was performed using the ClueGO plugin in Cytoscape to further elucidate the biological relevance of the hub genes. The Clue-GO network, including the hub genes, is shown in Figure 7D. The most significant group of pathways was that with Interleukin-10 pathway hub.

### 2.6. Violin Plot and ROC Curve Analysis

**Figure 8:**
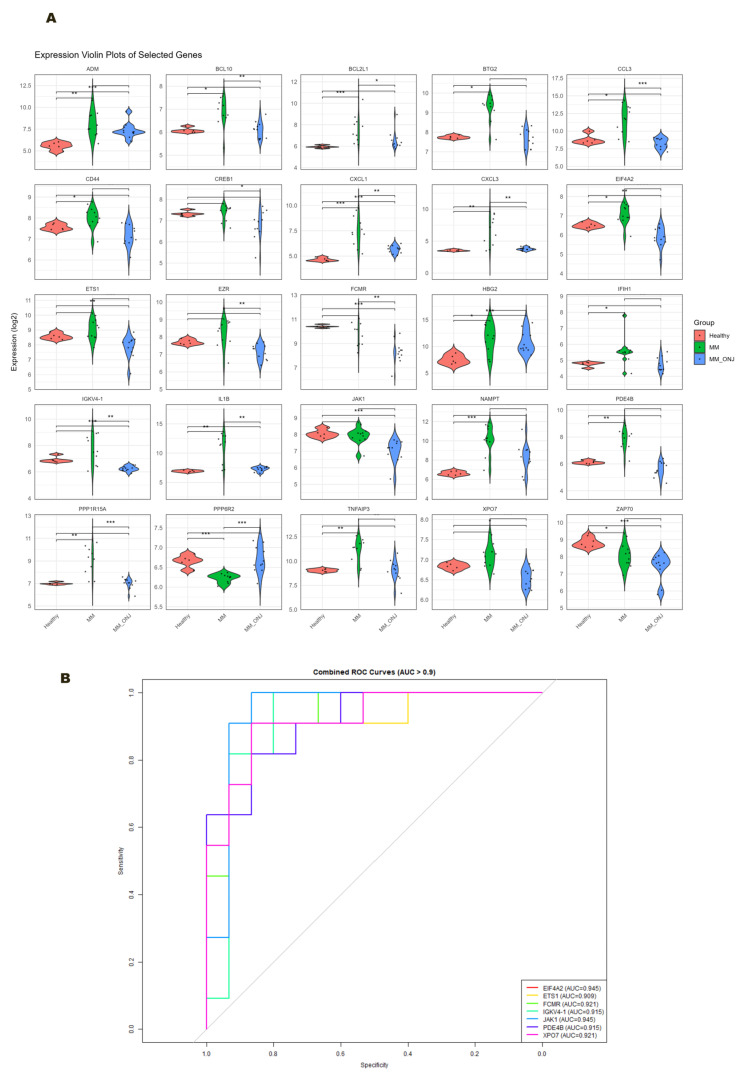
(**A**) Violin plots showing the log_2_ expression distribution of selected genes across the three groups: Healthy, MM, and MM_ONJ. Significance thresholds: *** *adj. p* < 0.001, ** *adj. p* < 0.01, * *adj. p* < 0.05. (**B**) ROC curves of genes with AUC > 0.9 and *adj. p* < 0.003.

RMA-normalized gene expression data were aggregated by gene symbol, and both the expression matrix and metadata were reshaped into long format for visualization. The expression patterns of 25 genes of interest across three experimental groups Healthy, MM, and MM_ONJ (*BCL2L1*, *ADM*, *BCL10*, *BTG2*, *CCL3*, *CD44*, *CREB1*, *CXCL1*, *CXCL3*, *EIF4A2*, *ETS1*, *EZR*, *FCMR*, *HBG2*, *IFIH1*, *IGKV4-1*, *IL1B*, *JAK1*, *NAMPT*, *PDE4B*, *PPP1R15A*, *PPP6R2*, *TNFAIP3*, *XPO7*, and *ZAP70*) were then visualized (Figure 8A). The assessment of differences between groups was conducted with the aid of the Wilcoxon rank-sum test, followed by false discovery rate (FDR) correction to account for multiple comparisons. This analysis revealed group-specific expression trends, with several genes showing statistically significant differences in expression between comparison groups Healthy vs. MM_ONJ and MM vs. MM_ONJ (Table 1). In addition, to assess the diagnostic potential of individual genes, ROC curve analysis was performed. Genes with an AUC > 0.9 and an *adj. p* < 0.003 (*EIF4A2*, *ETS1*, *FCMR*, *IGKV4-1*, *JAK1*, *PDE4B*, *XPO7*) were considered highly discriminative and selected for further investigation (Figure 8B).

### 2.7. Transcriptional Signature and Predictive Modeling of MRONJ-Associated Gene Expression

**Figure 9:**
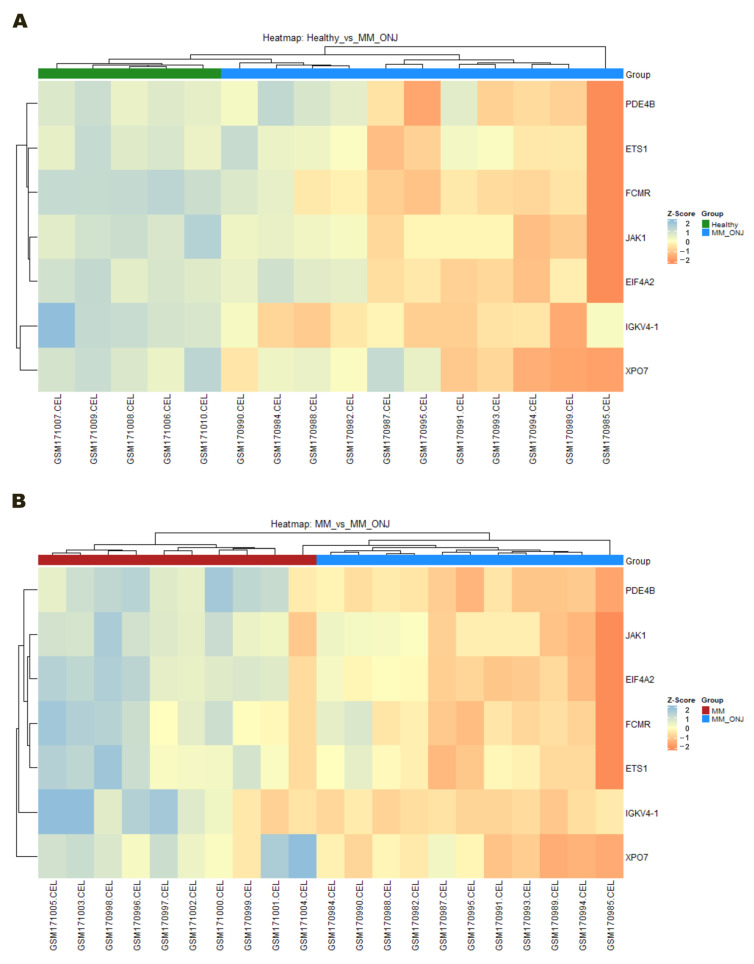
Heatmaps of candidate gene expressions in MRONJ comparisons. Z-score normalized expression levels of seven candidate genes (*PDE4B*, *ETS1*, *FCMR*, *JAK1*, *EIF4A2*, *IGKV4*-1, and *XPO7*) are shown across individual samples for (**A**) Healthy vs. MM_ONJ and (**B**) MM vs. MM_ONJ comparisons. Each column represents a sample, and each row a gene. Hierarchical clustering was applied to both genes and samples.

The generation of heatmaps enabled further evaluation of the expression patterns of candidate genes associated with osteonecrosis of the jaw. The comparisons used were Healthy vs. MM_ONJ and MM vs. MM_ONJ, with the use of z-score normalized expression values. In both comparisons, seven selected genes (*PDE4B*, *ETS1*, *FCMR*, *JAK1*, *EIF4A2*, *IGKV4-1*, and *XPO7*) demonstrated consistent upregulation in the MM_ONJ group relative to controls (Figure 9). In the Healthy vs. MM_ONJ heatmap, clear separation between groups was observed. The higher expression of all seven genes was demonstrated in MM_ONJ samples (Figure 9A). This trend also remained evident in the MM vs. MM_ONJ comparison, although with slightly reduced contrast (Figure 9B). The hierarchical grouping of samples demonstrated expression profiles that were specific to individual groups, thereby highlighting the discriminatory power of the genes that were selected. The most significant increase in expression was observed in the *PDE4B*, *FCMR*, *XPO7*, and *EIF4A2* genes in the MRONJ samples. These findings support the hypothesis that these genes may serve as potential biomarkers for MRONJ. Furthermore, they imply the presence of a distinct transcriptional signature associated with MRONJ development, separate from the transcriptional changes generally associated with MM.

The predictive accuracy of gene expression signatures was evaluated by minimizing bias resulting from small sample sizes and imbalanced groups. For this purpose, Firth’s logistic regression with reduced bias within nested CV was employed. The approach entailed the implementation of an outer loop, the function of which was to evaluate generalization performance. An inner loop was also incorporated, the purpose of which was to select features based on one-dimensional t-tests. In each iteration of the outer fold, the five genes exhibiting the lowest *p*-values were selected for model training.

**Figure 10:**
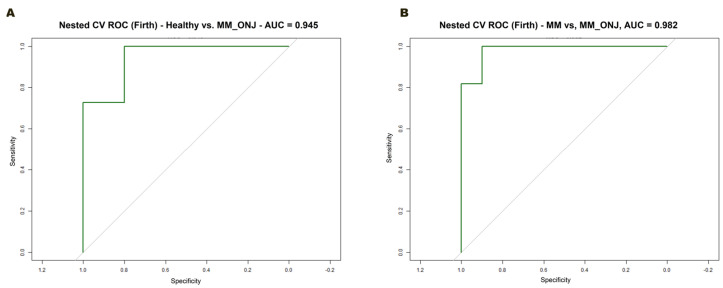
ROC curves from nested CV with Firth logistic regression. ROC curves were generated using out-of-fold predictions from a 5-fold nested cross-validation framework. In each outer fold, the top five genes with the lowest adjusted *p*-values (*adj. p* < 0.05) from the training set were selected and used to train a Firth-penalized logistic regression model. (**A**) ROC curve for the Healthy vs. MM_ONJ comparison (AUC = 0.945). (**B**) ROC curve for the MM vs. MM_ONJ comparison (AUC = 0.982).

Two binary classification tasks were evaluated: MM vs. MM_ONJ and Healthy vs. MM_ONJ (Figure 10). In a comparative analysis involving MM and MM_ONJ groups, an AUC of 0.982 was achieved (Figure 10A). This result represents an almost flawless distinction between these two different groups. A comparable result was observed in the comparison of the Healthy and MM_ONJ groups, where an AUC value of 0.945 was achieved (Figure 10B), which also indicates exceptional classification performance. These high AUC values suggest that MRONJ cases are molecularly distinct from both MM patients without MRONJ and from healthy controls. These findings collectively validate the strong discriminatory ability of the chosen gene signatures in detecting MRONJ.

**Figure 11:**
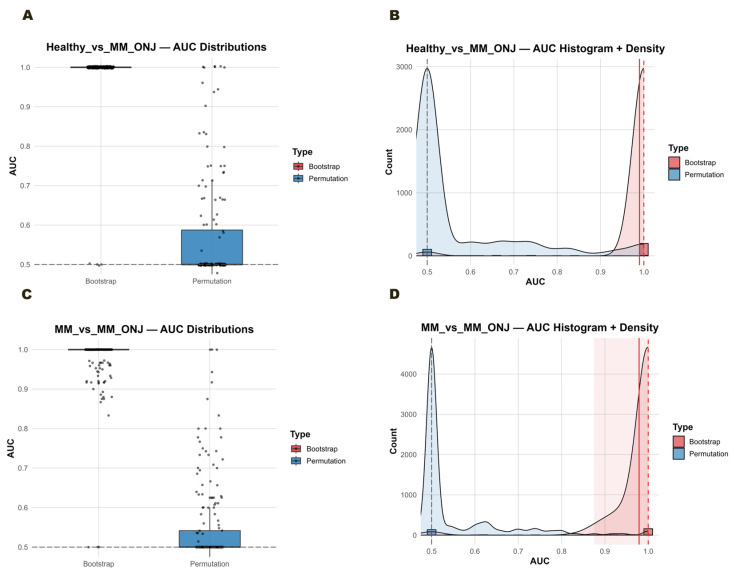
Discrimination performance for MRONJ classification using PCA + ridge logistic regression within a safe stratified 0.632+ bootstrap framework. (**A**) Healthy vs. MM_ONJ—Boxplot. Bootstrap-derived AUCs (red) and permutation-derived AUCs (blue) for LASSO-selected genes (*FCMR*, *IGKV4-1*, *JAK1*, *EIF4A2*). Bootstrap AUC median = 1.000 (95% CI: [1.000, 1.000]), empirical *p* = 0.0405, Cohen’s d = 3.99. Distributions are completely separated, indicating perfect classification. (**B**) Healthy vs. MM_ONJ—Histogram. The kernel density histograms demonstrate a complete separation between the bootstrap AUCs (red) and the permutation AUCs (blue), which confirms extremely strong discrimination. (**C**) MM vs. MM_ONJ—Boxplot. Bootstrap-derived AUCs (red) and permutation-derived AUCs (blue) for LASSO-selected genes (*PDE4B*, *EIF4A2*, *JAK1*, *XPO7*). Bootstrap AUC median = 1.000 (95% CI: [0.875, 1.000]), empirical *p* = 0.0152, Cohen’s d = 4.78. The results demonstrate strong discrimination, albeit with slightly greater variability compared to the results for Healthy vs. MM_ONJ. (**D**) MM vs. MM_ONJ—Histogram. The kernel density histograms show clear separation between bootstrap (red) and permutation (blue) AUCs, with only minimal overlap, confirming robust but slightly less perfect classification than Healthy vs. MM_ONJ.

We also evaluated the discriminatory ability of fixed LASSO-selected gene sets for Healthy vs. MM_ONJ and MM vs. MM_ONJ comparisons using PCA followed by ridge-penalized logistic regression within a safe stratified 0.632+ bootstrap framework. For each comparison, the dataset was restricted to the predefined four-gene panel, transformed to a samples × genes matrix, and analyzed across 200 bootstrap iterations with 200 matched stratified permutation tests to generate null AUC distributions. Model safety checks ensured adequate class representation in all resampling stages and folds. Healthy vs. MM_ONJ: Using the LASSO-selected panel (*FCMR*, *IGKV4-1*, *JAK1*, *EIF4A2*), the bootstrap-derived OOB mean AUC was 0.990, with a median of 1.000 and a 95% CI of [1.000, 1.000]. The 0.632+ mean AUC was 1.000. The empirical *p*-value was 0.0405, and the effect size (Cohen’s d) was 3.99, indicating an enormous separation between bootstrap and permutation distributions. The permutation AUC distribution was centered near the chance level (~0.5) with no overlap with the bootstrap distribution (Figure 11A,B). MM vs. MM_ONJ: For the LASSO-selected panel (*PDE4B*, *EIF4A2*, *JAK1*, *XPO7*), the bootstrap-derived OOB mean AUC was 0.979, with a median of 1.000 and a 95% CI of [0.875, 1.000]. The 0.632+ mean AUC was 0.991. The empirical *p*-value was 0.0152, and Cohen’s d was 4.78, again indicating an extremely large separation between distributions. While discrimination remained very strong, the bootstrap distribution showed slightly greater spread compared to the Healthy vs. MM_ONJ analysis, suggesting higher biological heterogeneity in this comparison (Figure 11C,D). Both gene panels achieved near-perfect classification performance under rigorous bootstrap evaluation. The complete or near-complete separation between bootstrap and permutation distributions, together with large effect sizes, confirms that these results are highly unlikely under the null hypothesis and robust to resampling variability. Across both comparisons, the complete separation between bootstrap and permutation distributions confirms that the models capture biologically meaningful differences in gene expression rather than artifacts of sampling or overfitting.

## 3. Discussion

In the present study, an integrative transcriptomic and machine-learning pipeline was implemented for the objective of identifying and validating systemic biomarkers of MRONJ in patients diagnosed with multiple myeloma. Through the combination of differential gene expression analysis, WGCNA, LASSO regression, bootstrap and permutation validation, and CytoHubba network ranking, we delineated molecular signatures implicating immune dysregulation, inflammatory signaling, and, as a hypothesis extension, maladaptive stress responses that may exacerbate bone necrosis.

### 3.1. Immune and Inflammatory Pathways in MRONJ

Our DEG and enrichment analyses revealed that the Healthy vs. MM_ONJ comparison exhibited consistent upregulation of translational and ribosomal machinery pathways (e.g., eukaryotic translation elongation, initiation, and rRNA processing) alongside suppression of neutrophil degranulation, antimicrobial peptide signaling, and defensin pathways. This pattern suggests an enhanced translational drive accompanied by weakened innate immune and antimicrobial responses, potentially compromising mucosal defense and jaw tissue resilience [21,22,23,24]. In contrast, analyses of MM vs. MM_ONJ comparison revealed an augmented state of adaptive immune activation, as indicated by increased regulation of T-cell signaling (TCR), FcεRI-mediated NF-κB activation, and mRNA cap-binding/eIF pathways, along with diminished regulation of keratinization, potassium channels, and epithelial barrier processes [25,26]. It is hypothesized that the aforementioned complementary characteristics of MRONJ imply a disturbance in immune-bone homeostasis, wherein uncontrolled immune stimulation is inadequately counterbalanced by protective innate and barrier mechanisms. Intersecting DEGs with WGCNA modules refined these findings to 40 hub module DEGs (Healthy vs. MM_ONJ) and 116 hub module DEGs (MM vs. MM_ONJ). Focusing on genes embedded within coherent co-expression networks rather than isolated DEGs improves biological interpretability and highlights modules likely reflecting systemic immunological perturbations associated with MRONJ susceptibility [27,28,29].

### 3.2. LASSO Selection and Model Validation

LASSO regression with ten-fold cross-validation distilled compact, interpretable gene panels. In Healthy vs. MM_ONJ, the λ_1_ₛₑ model retained five predictors—*FCMR*, *ZAP70*, *ADM*, *NAMPT*, and *HBG2*—whereas in MM vs. MM_ONJ, the selected set comprised *IGKV4-1*, *PPP6R2*, *XPO7*, *BTG2*, and *PDE4B*. As *HBG2* is a globin gene that is likely indicative of pre-analytical contamination, it was excluded from further analyses. Notably, the classifier exhibited consistent performance in the absence of *HBG2*, thereby confirming the biological validity of the results, which extend beyond the confines of technical artifacts. Complementary validation strategies reinforced these results. Nested cross-validation using Firth logistic regression achieved AUC = 0.945 for Healthy vs. MM_ONJ and AUC = 0.982 for MM vs. MM_ONJ. PCA + ridge logistic regression, embedded in a safe stratified 0.632+ bootstrap, yielded median bootstrap AUCs of 1.00 in both comparisons (Cohen’s d ≈ 3.99–4.78). Permutation null distributions were completely separated from bootstrap AUC distributions, indicating that discrimination was not driven by random variation. These findings collectively affirm the reproducibility and generalizability of the gene panels. While the model achieved AUC > 0.9 in internal validation, external cohort validation and tissue-level assays will be required to confirm the diagnostic utility of the proposed biomarkers. However, it is crucial to acknowledge that the bootstrap and permutation methods employed to assess the accuracy of the model’s performance are not inherently immune to bias. An AUC of 1.00 may reflect limited cohort diversity and should be interpreted cautiously.

### 3.3. Hub Network and Biological Interpretation

CytoHubba network analysis identified sixteen highly connected hub genes in both comparisons, including *JAK1*, *IL1B*, *TNFAIP3*, *ETS1*, *CXCL1*, and *CXCL3*. Many of these genes have well-described roles in cytokine signaling, NF-κB regulation, and immune-cell activation, which are central pathways in osteoimmunology. *JAK1* is a key component of several cytokine receptor systems (for example, IL-6 and γc cytokines) [30,31] and can influence osteoclast activity by modulating *RANKL* expression [32]. *TNFAIP3* (*A20*) acts as a negative regulator of NF-κB and helps maintain limits on inflammatory signaling and osteoclast differentiation [33]. *IL1B*, *CXCL1*, and *CXCL3* promote neutrophil recruitment and inflammatory cell movement [34,35]; these processes support mucosal healing but when prolonged may contribute to tissue injury. Persistent chemokine signaling in the oral mucosa is consistent with the non-healing ulcers seen in MRONJ. ETS1 is a transcription factor involved in immune-cell differentiation and inflammatory gene expression. It can also affect bone remodeling by increasing *RANKL* and *MMP* transcription in fibroblasts [36], linking inflammation with tissue breakdown and impaired repair [37]. Although direct evidence in MRONJ is limited, these mechanisms support their relevance to immune-bone interactions.

Overall, the network findings point to sustained cytokine and chemokine dysregulation in MRONJ, where unresolved inflammation may drive local tissue damage and impaired bone healing.

### 3.4. Hypothesis: Stress Responses as Co-Modifiers

Although immune dysregulation in MRONJ is well recognized [38,39], our enrichment analysis also pointed to changes in translation, ribosomal biogenesis, and eIF signaling. These findings are consistent with activation of the integrated stress response (ISR). We suggest that impaired stress-handling pathways—combining altered translational control, oxidative stress, and the unfolded protein response—may worsen immune-mediated tissue injury. There is mechanistic support for this idea. ISR signaling (*PERK* → *eIF2α* → *ATF4*) helps cells adapt to metabolic and oxidative stress. In osteoblasts, *ATF4* is required for normal matrix production and stress tolerance, and its disruption can limit osteogenesis and hinder bone repair [40,41]. Oxidative stress also promotes osteoclast differentiation and bone resorption [42] and increased reactive oxygen species have been shown to intensify bisphosphonate-associated necrosis in animal studies [43].

Considering these findings, we hypothesize that in patients with MM who are treated with antiresorptive agents, persistent immune activation and the reduced capacity to manage cellular stress, which are concomitant with the treatment, increase the susceptibility to jaw osteonecrosis. To validate this model, future research should assess ISR-related markers (e.g., phosphorylated *eIF2α* and *ATF4*-dependent genes), oxidative stress markers, and immune cell phenotypes in patient PBMCs and affected bone tissue.

### 3.5. Limitations and Future Directions

The present study has limitations. The modest sample size and limited clinical diversity of the dataset analyzed constrain the generalizability of the results. Although resampling and bias-reduction techniques (nested CV, Firth logistic regression, and 0.632+ bootstrap) were implemented to mitigate overfitting, external validation in larger, prospectively collected, and demographically heterogeneous cohorts remains essential to confirm reproducibility. PBMC transcriptomes serve as systemic surrogates for the jaw microenvironment. Thus, tissue-level validation of the identified biomarkers is essential to establish causality. Furthermore, the proposed stress-response hypothesis remains inferential and requires mechanistic experimentation.

The findings should therefore be viewed as exploratory. Future studies should include larger, prospectively collected cohorts and incorporate single-cell or spatial transcriptomics of jaw lesions to confirm cell-type-specific expression of the hub genes. We plan to validate our seven-gene MRONJ signature in a prospective multi-center study using independent patient cohorts recruited from collaborating institutions. Standardized protocols for sample collection and processing will be implemented to minimize inter-site variability. In parallel, we will perform targeted transcript quantification using the NanoString nCounter^®^ platform (either a Panel Plus–enhanced immune panel or a custom CodeSet) to confirm differential expression with a highly reproducible, amplification-free technology. These combined efforts will provide robust external validation and assess the clinical utility of the proposed biomarker panel in broader patient populations.

Preclinical experimentation involving the modulation of JAK—STAT, NF-κB, or ISR signaling in MRONJ animal models may facilitate the elucidation of causal pathways. Also, the integration of proteomic and phospho-signaling datasets has the potential to offer insights into post-transcriptional regulation and signaling activity.

Although PBMC transcriptomes reflect systemic immune and stress-related dysregulation, they may not fully capture the local molecular landscape of necrotic bone tissue. However, several hub genes identified here (*JAK1*, *TNFAIP3*, *ETS1*, and *CXCL* family members) have been independently reported as upregulated in MRONJ jawbone lesions or gingival tissues (e.g., [17,21]), suggesting partial correspondence between systemic and local expression profiles. In addition to tissue-level studies, salivary extracellular vesicles (EVs) represent an emerging, non-invasive source of molecular information, as their proteomic and transcriptomic cargo can mirror local inflammatory and osteolytic processes occurring in the oral cavity. Integrating salivary EV profiling with PBMC-based signatures may therefore help bridge systemic and site-specific pathology. Future work employing single-cell or spatial transcriptomics of jaw lesions, combined with EV-based molecular profiling, will be essential to establish cell-type-specific expression of these hub genes and clarify systemic-local signaling relationships in MRONJ.

### 3.6. Clinical Implications

The seven-gene signature (*PDE4B*, *ETS1*, *FCMR*, *JAK1*, *EIF4A2*, *IGKV4-1*, *XPO7*) offers a practical foundation for developing a multiplex PCR or digital droplet PCR (ddPCR) assay. Future translational steps will involve optimizing assay sensitivity and specificity, defining diagnostic thresholds based on ROC-derived cutoffs, and evaluating cost efficiency relative to current imaging-based screening. Integration with clinical predictors (e.g., duration of bisphosphonate therapy, comorbidities) may enable composite risk scores suitable for clinical decision support. Ultimately, panel refinement and validation could precede prospective evaluation in a biomarker-guided clinical trial.

The hub genes and molecular patterns identified here may have clinical relevance. A peripheral blood transcriptomic panel that includes *JAK1*, *TNFAIP3*, *CXCL1/3*, *ETS1*, and *FCMR* could potentially serve as a minimally invasive tool for assessing MRONJ risk before starting antiresorptive or antiangiogenic treatment. The implicated pathways—such as NF-κB, JAK–STAT, and chemokine signaling—also suggest opportunities for therapeutic modulation, for example, through cytokine inhibitors or JAK inhibitors, to limit inflammatory bone loss.

If the stress-response hypothesis is confirmed, treatments aimed at improving proteostasis or reducing oxidative stress (such as antioxidants or ISR-modulating agents) may complement current preventive approaches. Ultimately, biomarker-guided patient selection combined with pathway-targeted intervention could lower MRONJ risk and improve treatment safety in oncology and osteoporosis care.

## 4. Materials and Methods

### 4.1. Data Resource Collection

Gene expression microarray data from patients with osteonecrosis of the jaw [44] were obtained from the Gene Expression Omnibus (GEO) database (accession GSE7116) (National Institutes of Health, Bethesda, MD, USA; http://www.ncbi.nlm.nih.gov/geo, accessed on 10 June 2025) using the R programming language. The dataset was obtained utilizing the keywords ‘osteonecrosis of the jaw’, ‘*Homo sapiens*’, and ‘GPL570 platform’. It comprises transcriptional profiles of peripheral blood mononuclear cells (PBMCs) from 11 multiple myeloma patients with MRONJ (MM_ONJ: GSM170982.CEL, GSM170984.CEL, GSM170985.CEL, GSM170987.CEL, GSM170988.CEL, GSM170989.CEL, GSM170990.CEL, GSM170991.CEL, GSM170993.CEL, GSM170994.CEL, GSM170995.CEL), compared with 10 age-matched multiple myeloma controls (MM: GSM170996.CEL, GSM170997.CEL, GSM170998.CEL, GSM170999.CEL, GSM171000.CEL, GSM171001.CEL, GSM171002.CEL, GSM171003.CEL, GSM171004.CEL, GSM171005.CEL) and five healthy volunteers (Healthy: GSM171006.CEL, GSM171007.CEL, GSM171008.CEL, GSM171009.CEL, GSM171010.CEL). Patients (aged 57–81 years) had received pamidronate (n = 3), zoledronate (n = 4), or both (n = 4) for an average of 38.7 months. The GPL570 platform (Affymetrix GeneChip Human Genome U133 Plus 2.0 Array, Affy-metrix, Santa Clara, CA, USA) was used.

### 4.2. Data Normalization and Differential Expression Analysis

The raw microarray data were processed with the Affy package (v 1.68.0; Bio-conductor) in R (v 4.4.2; CRAN). The dataset was obtained from the GEO database through the GEOquery package (v 2.74.0). To ensure the acquisition of high-quality, standardized expression data, a series of procedures were implemented. Background correction and normalization were first performed using the Robust Multi-array Average (RMA) algorithm, followed by quantile normalization. The analysis of differential expression was performed utilizing the limma package (v 3.62.2). The lmFit and eBayes functions were utilized to model expression data and calculate moderated statistics for differential gene expression across groups. A comparative analysis was performed involving patients diagnosed with multiple myeloma and MRONJ (MM_ONJ) alongside two control groups: patients with multiple myeloma without MRONJ (MM) and healthy individuals (Healthy). A cutoff criterion of absolute log2-fold change (|log_2_FC|) > 1 and *p* < 0.05 was utilized to identify significantly differentially expressed genes (DEGs). The results were visualized using a heatmap that displayed the top 30 DEGs according to their (by |log_2_FC|) values, alongside volcano plots illustrating both upregulated and downregulated genes. All visualizations were generated using the ggplot2 (v 4.0.0) and pheatmap (v 1.0.13) packages in the R programming environment.

### 4.3. Gene Set Enrichment Analysis

A Gene Set Enrichment Analysis (GSEA) was performed on pre-ranked gene lists using the clusterProfiler package (v 4.14.6) in R or the GSEA (v 4.3.3) desktop application (Broad Institute) using the Kyoto Encyclopedia of Genes and Genomes (KEGG) and Reactome pathway curated databases.

To evaluate the statistical significance of the observed results, enrichment scores were computed utilizing a Kolmogorov–Smirnov-like statistic with 100,000 permutations. This study identified pathways with an adjusted *p*-value (*adj. p* < 0.05) as significantly enriched. The ten pathways identified as significantly overexpressed, and the ten pathways identified as significantly underexpressed were subsequently ranked based on their Normalized Enrichment Score (NES). To enhance understanding, these rankings were later illustrated through bar plots, created with the R statistical software (v 4.4.2).

### 4.4. Weighted Gene Co-Expression Network Analysis

A Weighted Gene Co-expression Network Analysis (WGCNA) [45] was carried out to identify modules of co-expressed genes that are associated with clinical traits (WGCNA, v1.73). The raw microarray data underwent a series of standard procedures, including normalization using the Robust Multi-array Average (RMA) algorithm and log_2_-transformation of expression values. The low-variance genes were filtered out by keeping the top 75% of the most variable probes. The quality of the samples was then assessed using hierarchical clustering, a method that detects and excludes outliers.

The selection of a soft-thresholding power (β) was made using the scale-free topology criterion (R^2^ ≥ 0.85), with the fit indices being computed over a range of powers. An unsigned network was constructed using the blockwiseModules function. To assess connectivity among genes, the adjacency and topological overlap matrices (TOM) were calculated. Initially, modules were defined by dynamic tree cutting. Subsequently, they were merged if their eigengene dissimilarity was less than 0.25 (i.e., eigengene correlation > 0.75). The mergeCloseModules function was used to carry out these merges. Module eigengenes (MEs), which correspond to the initial principal component of each module, were correlated with binary clinical traits (Healthy vs. MM_ONJ, MM vs. MM_ONJ) employing Pearson’s correlation. Subsequently, statistically significant module-trait associations (*p* < 0.05) were represented visually via labeled heatmaps. Probe identifiers were mapped to gene symbols using the hgu133plus2.db annotation package.

### 4.5. Integrated Hub Gene Identification: LASSO and CytoHubba

To determine dominant hub genes, two distinct approaches were used: Least Absolute Shrinkage and Selection Operator (LASSO) regression and CytoHubba-based protein–protein interaction (PPI) network analysis. These analyses were conducted separately for the Healthy vs. MM_ONJ and MM vs. MM_ONJ comparisons. LASSO was utilized for the selection of predictive genes from expression data, whereas Cyto-Hubba identified topological hub genes based on network connectivity.

#### 4.5.1. LASSO Regression

The gene expression data were divided into subsets and normalized prior to modeling. For each comparison, a penalized logistic regression model was fitted using the glmnet package (v 4.1.10) in R, and cross-validation (CV) via cv.glmnet was used to determine the optimal penalty parameter (lambda.min). Genes with non-zero coefficients at lambda.min were identified as LASSO-derived hub genes. When only a single predictor remained, standard logistic regression was applied instead.

#### 4.5.2. CytoHubba

Protein–protein interaction networks were constructed and analyzed using Cytoscape (v 3.10.2) [46]. The STRING database was used as the source for interaction data, and the StringApp (v 2.1.1) [47] extension facilitated data import and visualization. The CytoHubba (v 0.1) plugin [48] was employed to identify hub genes using all available scoring algorithms (e.g., MCC, Degree, EPC). To this aim, an UpSet plot was generated to identify genes that appeared in at least three of the ranking methods, thus highlighting consistently top-ranked hub candidates.

### 4.6. Gene Ontology and Pathway Enrichment

Gene Ontology (GO) enrichment analysis was performed using the BiNGO (v 3.0.5) extension [49] in Cytoscape to evaluate the overrepresentation of hub genes within hierarchical GO categories. The investigation was executed across three domains: biological process (BP), molecular function (MF), and cellular component (CC), with the objective of elucidating the functional roles and regulatory context of the identified gene sets. Multiple testing correction was executed by implementing the Benjamini–Hochberg false discovery rate methodology, employing a significant threshold of *p* < 0.05. Parallel to this, pathway enrichment analysis was conducted using the ClueGO/CluePedia (v 2.5.10) [50] plugin in Cytoscape. This approach comprised the integrated interrogation of both KEGG and Reactome pathway databases, with the intention of further elucidating the biological relevance of the hub genes within the framework of recognized signaling and metabolic pathways.

### 4.7. Violin Plot and ROC Curve Analysis

RMA-normalized gene expression data were aggregated by gene symbol, and both expression values and corresponding metadata were reshaped into a long format for the purpose of further analysis and visualization. The violin plots were constructed with the aid of the ggplot2 (v 4.0.0) and ggpubr (v 0.6.1) packages in R. Group differences were assessed using the Wilcoxon rank-sum test, followed by false discovery rate (FDR) correction to control for multiple comparisons. The discriminatory power of individual genes was evaluated using receiver operating characteristic (ROC) analysis implemented using the pROC package. Genes that demonstrated an area under the curve (AUC) > 0.9 and an adjusted *p*-value < 0.003 were considered to possess significant discriminatory power and were therefore selected for further investigation.

### 4.8. Nested Cross-Validation and Firth Logistic Regression

A nested CV framework was applied for evaluation of the predictive power of gene expression profiles to distinguish between MM_ONJ and control groups (Healthy or MM). The outer loop was used exclusively for model evaluation, while the inner loop performed feature selection. In each outer fold, statistical filtering was applied to the training data to identify significantly differentially expressed genes. For this, a two-sample t-test followed by false discovery rate (FDR) adjustment was used. Then, as predictors, the top five genes with the lowest adjusted *p*-values (*adj. p* < 0.05) were selected. The logistf (v 1.26.1) package enabled us to employ these features to train Firth-penalized logistic regression models to address the challenges posed by limited data samples and mitigate potential biases in parameter estimation. Subsequently, an evaluation of the model’s performance was conducted by employing out-of-fold predictions from the outer CV loop. The ROC curves were generated from these predictions using the pROC (v 1.19.0.1) package. The AUC was calculated for each comparison. To facilitate this comparison, ROC curves were generated for the Healthy vs. MM_ONJ and MM vs. MM_ONJ groups. The AUC values were reported for each curve.

### 4.9. PCA + Ridge Regression with Safe Stratified 0.632+ Bootstrap

A safe stratified 0.632+ bootstrap framework was implemented to reduce optimism bias in AUC estimation and to assess statistical significance against the null distribution. The pipeline combined Principal Component Analysis (PCA) with ridge-penalized logistic regression. For each comparison (Healthy vs. MM_ONJ and MM vs. MM_ONJ), the dataset was restricted to the fixed four-gene LASSO-selected panel specific to that comparison. Genes were intersected with available expression features, and the resulting expression matrix was transposed to samples × genes format. A total of 200 bootstrap iterations were performed. In each iteration, stratified sampling with replacement selected 63.2% of the samples for the training set, leaving the remainder as out-of-bag (OOB) test samples. Safety constraints included the following: 1. Both outcome classes present in both training and test sets, 2. ≥2 samples per class in the training set before inner cross-validation, 3. Inner 3-fold stratified cross-validation with ≥2 samples per class in each fold. PCA was fitted only on the training set, retaining the minimum number of components required to explain ≥95% of variance, capped at three components. These components were used to train a ridge-penalized logistic regression model (cv.glmnet, α = 0). A safe CV routine was used, defaulting to “deviance” as the type.measure if “auc” was unstable. The following AUC metrics were recorded: Apparent AUC (training set), OOB AUC (test set), and 0.632+-corrected AUC, calculated with error = 1 − AUC and no-information error = 0.5. To assess significance, 200 stratified permutation iterations were performed. In each iteration, outcome labels were randomly shuffled while preserving the feature matrix, and the same PCA + ridge pipeline was applied to generate a null AUC distribution. The empirical *p*-value was computed as the proportion of permutation AUCs greater than or equal to the median 0.632+ AUC from the bootstrap. The 95% confidence interval (CI) for OOB AUCs was calculated from empirical quantiles. Cohen’s d effect size was computed between bootstrap and permutation AUC distributions.

## 5. Conclusions

In summary, the integrative pipeline that was utilized in this study yielded a convergent molecular portrait of MRONJ. This condition is driven by immune-inflammatory dysregulation, with stress responses hypothesized as critical modifiers. These findings provide both biomarker hypotheses and mechanistic insights that align with the intention to link molecular discovery and translational medicine to facilitate biomarker-guided risk prediction in MRONJ.

## Figures and Tables

**Table 1:** Top significantly differentially expressed genes based on *adj. p* (FDR < 0.05) for comparisons: Healthy vs. MM_ONJ, MM vs. MM_ONJ.

Gene	Comparison	Raw *p*-Value	Adjusted *p*-Value
*ADM*	Healthy vs. MM_ONJ	0.000458	0.002938
*CXCL1*	Healthy vs. MM_ONJ	0.000458	0.002938
*FCMR*	Healthy vs. MM_ONJ	0.000458	0.002938
*IGKV4-1*	Healthy vs. MM_ONJ	0.000458	0.002938
*JAK1*	Healthy vs. MM_ONJ	0.000458	0.002938
*ZAP70*	Healthy vs. MM_ONJ	0.000458	0.002938
*HBG2*	Healthy vs. MM_ONJ	0.000916	0.003434
*EIF4A2*	Healthy vs. MM_ONJ	0.008700	0.017170
*ETS1*	Healthy vs. MM_ONJ	0.008700	0.017170
*EZR*	Healthy vs. MM_ONJ	0.013278	0.021189
*PDE4B*	MM vs. MM_ONJ	0.000011	0.000851
*EIF4A2*	MM vs. MM_ONJ	0.000068	0.002552
*BTG2*	MM vs. MM_ONJ	0.000170	0.002938
*CCL3*	MM vs. MM_ONJ	0.000550	0.002938
*JAK1*	MM vs. MM_ONJ	0.000550	0.002938
*PPP6R2*	MM vs. MM_ONJ	0.000550	0.002938
*XPO7*	MM vs. MM_ONJ	0.000255	0.002938
*ETS1*	MM vs. MM_ONJ	0.000788	0.003111
*PPP1R15A*	MM vs. MM_ONJ	0.000788	0.003111
*CD44*	MM vs. MM_ONJ	0.001100	0.003750

## Data Availability

The data presented in this study are available in NCBI GEO database at https://www.ncbi.nlm.nih.gov/geo/query/acc.cgi?acc=GSE7116, accessed on 10 June 2025, reference number GSE7116. These data were derived from the following resources available in the public domain: https://www.ncbi.nlm.nih.gov/geo/query/acc.cgi?acc=GSE7116.

## Supplementary Materials

The following supporting information can be downloaded at: https://www.mdpi.com/article/10.3390/ijms262411788/s1.
